# Supplementary material for: Characterization and applications of evoked responses during epidural electrical stimulation
Source: Bioelectron Med. 2023 Feb 28;9:5. doi: 10.1186/s42234-023-00106-5 (PMC9976490; doi:10.1186/s42234-023-00106-5)
Supplement: Supplementary file 1 — Additional file 1: Supplementary Fig. 1. Lead movement for animal subject S3 shown in Fig. 5. (A) Caudal movement of stimulation lead. (B) Lateral movement of stimulation lead. Supplementary Fig. 2. X-Ray images of location used for stimulation. [file 42234_2023_106_MOESM1_ESM.docx]

**Supplementary Material**

**
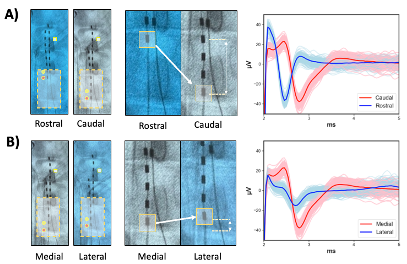
**

**Supplementary Figure 1:** Lead movement for animal subject S3 shown in Fig5. (A) Caudal movement of stimulation lead. (B) Lateral movement of stimulation lead.

**
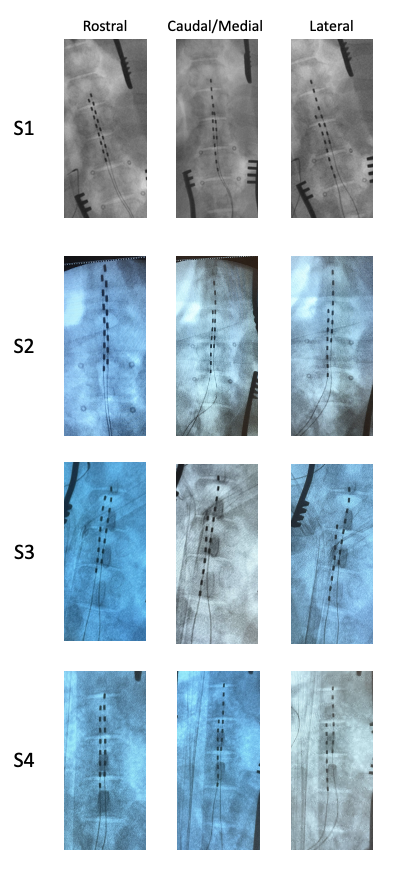
**

**Supplementary figure 2:** X-Ray images of location used for stimulation.
